# Tiny Vehicle Detection for Mid-to-High Altitude UAV Images Based on Visual Attention and Spatial-Temporal Information

**DOI:** 10.3390/s22062354

**Published:** 2022-03-18

**Authors:** Ruonan Yu, Hongguang Li, Yalong Jiang, Baochang Zhang, Yufeng Wang

**Affiliations:** 1 School of Electrical and Information Engineering, Beihang University, Beijing 100191, China; yrn_1126@buaa.edu.cn; 2 Unmanned System Research Institute, Beihang University, Beijing 100191, China; lihongguang@buaa.edu.cn (H.L.); wyfeng@buaa.edu.cn (Y.W.); 3 School of Automation Science and Electrical Engineering, Beihang University, Beijing 100191, China; bczhang@139.com

**Keywords:** mid-to-high altitude UAV images, tiny object detection, visual attention, spatial-temporal information

## Abstract

Mid-to-high altitude Unmanned Aerial Vehicle (UAV) imagery can provide important remote sensing information between satellite and low altitude platforms, and vehicle detection in mid-to-high altitude UAV images plays a crucial role in land monitoring and disaster relief. However, the high background complexity of images and limited pixels of objects challenge the performance of tiny vehicle detection. Traditional methods suffer from poor adaptation ability to complex backgrounds, while deep neural networks (DNNs) have inherent defects in feature extraction of tiny objects with finite pixels. To address the issue above, this paper puts forward a vehicle detection method combining the DNNs-based and traditional methods for mid-to-high altitude UAV images. We first employ the deep segmentation network to exploit the co-occurrence of the road and vehicles, then detect tiny vehicles based on visual attention mechanism with spatial-temporal constraint information. Experimental results show that the proposed method achieves effective detection of tiny vehicles in complex backgrounds. In addition, ablation experiments are performed to inspect the effectiveness of each component, and comparative experiments on tinier objects are carried out to prove the superior generalization performance of our method in detecting vehicles with a limited size of 5 × 5 pixels or less.

## 1. Introduction

With the continuous development of computer vision technology and the improvement of commercial UAV platforms, UAVs have been widely used in various scenarios such as traffic detection, agriculture and forestry, and disaster prevention and relief. Due to its flight characteristics of high altitude and long duration, the information collected by mid-altitude UAVs, such as graphics, videos, and spectrum, is becoming increasingly abundant. Moreover, UAVs can perform tasks in hazardous areas and bad weather conditions with efficient data acquisition capability, playing an increasingly important role in civil and military fields [1,2].

Object detection, one of the most important tasks in computer vision, aims to identify different types of objects and mark their locations. With the development of deep learning techniques, as well as the establishment of high-quality UAV image datasets [3,4,5,6,7], vehicle detection in UAV images has become a research hotspot in recent years to accurately locate and identify vehicle targets, which is of great significance for traffic monitoring, urban planning, search and tracking, and other practical applications.

Remote shooting from long distances leads to high background complexity of images and small size of objects. However, small object detection among a complex background presents a challenging problem to be solved in the field of image analysis and processing. There are two typical ways to define small objects. In the COCO dataset [8], objects smaller than 32 × 32 pixels are considered as small objects. According to the International Society of Optical Engineering (SPIE), an object is defined as small if its size is less than 0.12% of the original image [9]. Under the mid-to-high altitude conditions of long-distance and oblique observation to the ground, the UAV image covers a wide range of area with a large number of pixels, while the object of interest usually occupies only several hundred or even dozens of pixels, which is far less than 0.12% of the image. Therefore, for mid-to-high altitude imaging platforms, objects with less than 10 × 10 pixels in the image are defined as tiny objects. 

Existing detection algorithms for small objects can be divided into two main categories: traditional image processing and deep learning methods, which have certain limitations for tiny object detection. Traditional image processing methods are mostly adopted in infrared small object detection for infrared search and rescue systems [10,11], such as morphology-based top-hat algorithm [12,13], wavelet transform [14,15], and visual saliency detection [16,17,18]. However, traditional manual-designed features suffer from insufficient representation and poor adaptability to complex backgrounds, which hinders their applications to visible images with complex backgrounds. Deep learning-based methods [19,20] achieve excellent performance on general datasets but low accuracy for tiny objects with finite pixels, whose feature is insufficient and even weakened after then common down-sampling operations in DNNs. Moreover, the training of the DNNs requires a large amount of annotated data that entails significant time and effort. Nevertheless, for the mid-to-high altitude UAVs, there is currently a lack of such large-scale annotated image datasets for the tiny object detection task.

**Figure 1:**
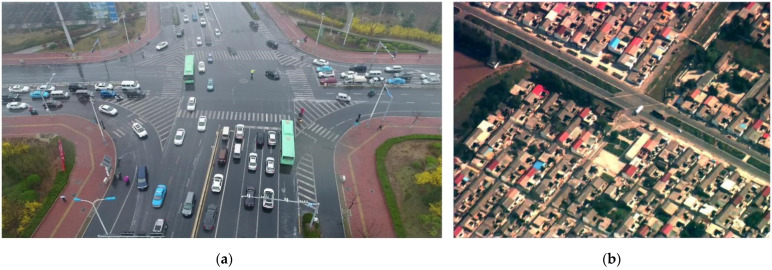
Comparison of UAV images at different heights. (**a**) An example of the low altitude UAV image dataset UAVDT [7], and (**b**) is an example of the mid-to-high altitude UAV image we established.

In this paper, a series of video sequences are collected to establish a mid-to-high altitude UAV images dataset. Different from low-altitude UAV images, the images collected by us are fuzzy with low contrast. The comparison of UAV images at different heights is shown in Figure 1. It can be found that objects in our image are smaller than in the low altitude image, and the background interference is larger, which further increases the difficulty of object detection.

To address the above problems, this paper develops a multi-stage tiny vehicle detection framework (MTVD) for mid-to-high altitude UAV images based on visual attention and spatial-temporal information. The proposed method takes advantage of both deep networks and traditional methods with a progressive integration of them. Considering the strong dependence relationship between the road and vehicles in the ground scene, the road areas in the image are extracted by segmentation networks to suppress the interference of complex backgrounds. Then tiny vehicles on the road are detected by the improved RSS [21] algorithm that incorporates stability region and saliency detection to strengthen the visual attention information. The motion information of vehicles is further employed to enhance the detection results with spatial-temporal confidence.

The contributions of this work are summarized as follows:

(1) We propose a multi-stage tiny vehicle detection framework with deep segmentation and traditional detection components to solve the problem that existing traditional methods have difficulty in detecting tiny objects from complex backgrounds.
(2) We improve the RSS algorithm with both visual attention and spatial-temporal information, termed as STRSS, to assist the detection procedure by designing a locally weighted saliency decision, which can remove the false alarm and increase the detection precision.
(3) The proposed method achieves effective detection of tiny vehicles under complex backgrounds with an F1 score of 78.32%. Moreover, our method is not limited by the size of the objects, and comparative experimental results show that it outperforms the advanced DNNs-based method for extremely tiny object detection with pixels smaller than 5 × 5.

This paper is organized as follows. Section 2 summarizes relevant literature, and Section 3 introduces the proposed methods of tiny object detection in detail. In Section 4, the dataset, evaluation metrics, and experimental details and results are presented. The conclusion is presented in Section 5.

## 2. Related Work

In this section, several traditional small object detection methods commonly for infrared weak and small object detection are introduced, including single-frame detection and moving object detection. In addition, some algorithms based on DNNs are also mentioned.

### 2.1. Single Frame Detection

Single frame detection is suitable for imaging scenes with uniform backgrounds. Classical methods include maximum mean or maximum median filter [22], morphology-based top-hat algorithm [12,13], wavelet transform [14,15].

In recent years, algorithms based on human visual attention mechanisms have achieved good performance which uses the difference between object and background to perform background suppression and noise removal, to find the regions of objects. Laplacian of Gaussian (LOG) filter [16] smooths the image through a Gaussian filter and then uses a Laplacian filter to make the image contour clearer. Wang et al. proposed an automatic detection algorithm based on visual attention, using the Difference of Gaussian (DOG) filter [17] to generate saliency maps for purpose of enhancing the region of the object and eliminating large areas of bright background. Achanta et al. [18] proposed a frequency tuning method for salient region detection, which uses the features of color and brightness to estimate the contrast between center and surrounding, so as to obtain the saliency map.

Since the object is locally uniform and has significant intensity differences from the surroundings, J. Matas proposed the Maximally Stable Extremal Region (MSER) [23] algorithm to perform binary segmentation of gray image by using continuous thresholds. In the process of changing the threshold, the region with the smallest area change is extracted as the maximally stable extremal region, to distinguish the object from the surrounding background. RSS [21] combines stability region detection and saliency detection to achieve small object detection in color images under simple background, but there are still problems of false alarms and missing objects.

### 2.2. Moving Object Detection

Moving object detection mainly utilizes the spatial-temporal continuity of the image sequence to improve detection accuracy by introducing more dynamic information. Spatial-temporal continuity means that the trajectory of the object in adjacent frames is continuous and the appearance change of it also has continuity in time. The method of detection-before-trace (DBT) is usually adopted. Firstly, single-frame detection is carried out on the image sequence to obtain the candidate object, and then the candidate is judged by the motion characteristics and other prior information to detect the real object.

Background estimation method [24,25] detects moving objects by modeling the background image and forming the difference between the original image and the background image, and it is only applicable to imaging sequences with static backgrounds. The frame difference method [26,27] obtains the moving object by forming a difference between several frames that are continuous in time or have a certain interval. 

Optical flow [28,29] is a classical motion estimation method, which calculates the motion field through the optical flow characteristics of the moving object over time, and then determines the motion trajectory of the object according to the motion field. Multi-frame energy accumulation [30,31] averages the preprocessed multi-frame images, so that the energy of moving small objects can be accumulated, while the random noise accumulation is slow, and ultimately distinguishes them. However, the background noise cannot be distinguished.

The basic idea of pipeline filtering [32,33,34] is that in a continuous N-frame image sequence, the position of the candidate object in the previous frame is the center of the pipeline, and the maximum distance that the object can move between frames is the radius of the pipeline. The candidate object detected M times in the pipeline is considered as the real object. Li et al. [32] improves the traditional pipeline filtering and proposed the adaptive pipeline filtering algorithm. The center and radius of the pipeline filter are updated adaptively according to the motion change of the target, so this algorithm has strong robustness to noise interference and target motion variation.

In summary, traditional object detection methods have made good progress in infrared weak and small object detection through visual attention mechanisms. In the task of moving object detection, the spatial-temporal continuity of the image sequence is used to introduce more information to improve detection accuracy. However, the above methods are suitable for scenes with uniform and simple backgrounds and are difficult to apply to tiny object detection task under complex backgrounds of visible images for mid-to-high altitude platforms.

### 2.3. DNNs-Based Small Object Detection

In recent years, object detection based on DNN has become mainstream. Most of the existing small object detection algorithms propose some improvement or optimization strategies based on the general detectors. Multi-scale feature fusion [35,36] and context information enhancement [37,38] are used to generate high-quality feature representation, so as to improve the robustness of the model to multi-scale objects. There are also certain methods to improve the detection performance of small objects such as designing appropriate anchor size [39,40], introducing visual attention mechanism [41,42], and data augmentation [43,44]. Recently, transformer technology has been gradually applied to computer vision, which provides a new solution to the problem of small object detection [45,46]

In the field of video object detection, the prediction effect of the current frame is mainly enhanced by using the characteristics or detection results of other frames [47,48,49]. SpotNet [50] trained foreground/background segmentation as well as object detection jointly via a multi-task learning approach to direct attention towards objective areas. The labels of foreground/background segmentation are produced by applying background subtraction or optical flow methods to video sequences.

DNNs extract different levels of features through multi-level structure, but down-sampling will gradually weaken the feature information of small objects. The proposed method avoids multi-layer feature extraction, and the detection effect is independent of the object size.

## 3. Materials and Method

In this section, the general framework of our method is shown in Section 3.1, RSS is briefly introduced in Section 3.2, and the details of each part of the proposed algorithm are presented in Section 3.3, which represents the key points of our improvement.

### 3.1. General Framework

**Figure 2:**
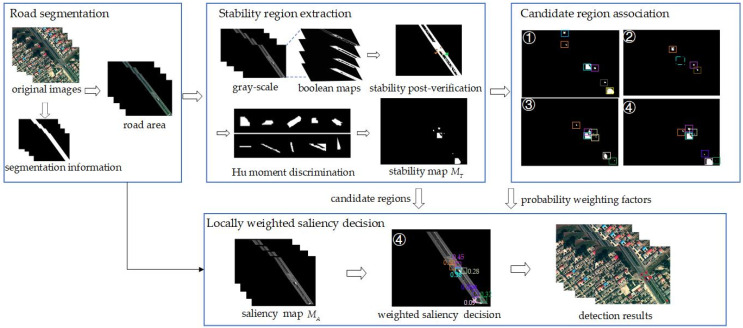
The framework of our method. Firstly, the original image and the segmentation information are combined by multiplication to generate the road area. Then the processed image is sent to the object detection network for stability region extraction, including gray-scale image conversion, multi-level threshold binarization segmentation and other steps to obtain the candidate regions of vehicles and a stability map. Then, we perform candidate region association based on the relative position relationship and appearance similarity of candidate regions in different frames to generate probability weighting factors. Both candidate regions and probability weighting factors are used for locally weighted saliency decision to remove false candidate regions(such as the pink detection box with score of 0.09 in the forth image) and obtain final detection results.

For mid-to-high altitude UAV images, this paper proposes a multi-stage tiny vehicle detection method. The overall process is shown in Figure 2. Firstly, the roads are extracted from the original UAV image by the deep semantic segmentation network. Then, according to the road area, we extract the stability regions and screen to obtain the candidate regions of vehicles. Next, on the basis of spatial-temporal continuity and appearance similarity, the candidate regions of the same vehicle in different frames are associated to generate a probability weighting factor, which represents the probability of the occurrence of each object in this period. Finally, the authenticity of the candidate region is judged by the locally weighted saliency detection score. If the saliency score is greater than the threshold, it is determined as the real object, otherwise, it is a false alarm.

### 3.2. RSS Algorithm

RSS [21] is a small object detection algorithm of color images that combines regional stability and saliency. It mainly includes three parts: stability region extraction, saliency detection, and integration of stability and saliency results.

#### 3.2.1. Regional Stability

In view of the fact that the object in the UAV image, especially the tiny vehicle, generally has the mass effect and exhibits clustering specialty. The shape of the object is relatively regular and there is a conspicuous gray difference between the object and the surroundings so that it can be regarded as a spot in the uniform background. These characteristics are used to construct the object/background priors and obtain the candidate region of the vehicle based on the stability region detection. The main steps are as follows.

(1) Multi-level threshold segmentation

Since the geometric and statistical invariance of objects during binarization, the multi-level threshold segmentation is applied to extract the stability region of images.

Firstly, the three-channel color image is converted into a grayscale image, then a set of thresholds with a grayscale range of [0, 255] and a step size of δ are used to perform binary segmentation on the grayscale image to obtain several sets of connected regions and their corresponding five structure descriptors. The five structural descriptors of each connected area in the binary image are: (1) the number of pixels |r|; (2) geometric center point *c*; (3) Minimum outer rectangle *b*; (4) filling rate *f*; (5) aspect ratio *a*.

(2) Connected regions clustering

To describe the similarity between two adjacent connected regions *u* and *v*, four stability measure criteria are defined as follows:

(a) area variation: $D_{r}(u,v)=||u|-|v||$;
(b) center distance: $D_{c}(u,v)=||c_{u}-c_{v}||$;
(c) fill rate difference: $D_{f}(u,v)=\frac{\max(f_{{}_{u}},f_{v})}{\min(f_{{}_{u}},f_{v})}$
(d) aspect ratio difference: $D_{a}(u,v)=\frac{\max(a_{{}_{u}},a_{v})}{\min(a_{{}_{u}},a_{v})}$

Taking the Euclidean distance between the geometric center points of each connected region as the constraint, the connected regions obtained by binary segmentation are clustered through the spatial relationship between them. It is judged whether the center distance of two adjacent regions is less than the threshold. If so, the regions are in the same cluster.

The threshold $\Delta_{c}$ is defined as:

(1) $$\Delta_{c}=\frac{\min^{2}(b_{r_{i},w},b_{r_{j},w})+\min^{2}(b_{r_{i},h},b_{r_{j},h})}{4}$$

where the subscripts $b_{r_{i},w}$, $b_{r_{i},h}$ indicate the width and height of the regions $r_{i}$.

(3) Stability region post-verification

Fill rate difference and aspect ratio difference indicate the degree of difference in the appearance of two connected areas. The smaller the difference between them, the more stable the corresponding target region is. Therefore, among four connected regions corresponding to the minimum difference of filling rate and aspect ratio, we select the region with the largest number of pixels as the maximally stable region of the object and take the minimum enclosing rectangle box of it as the optimal sub-image containing only one object. Then, the Otsu method [51] is used to segment the optimal sub-image to maximize the variance between the object and the background to obtain the optimal threshold ϑ. Next, the second post-verification of regions is performed by taking two thresholds ϑ-δ/2, ϑ+δ/2 to segment the optimal sub-image again and obtain the corresponding segmentation result $R^{\vartheta_{-}}$, $R^{\vartheta_{+}}$. If the area change $D_{r}(R^{\vartheta_{-}},R^{\vartheta_{+}})$ is less than the threshold $\Phi_{r}$, the region is determined to be a stable region; otherwise, it is directly discarded.

The threshold $\Phi_{r}$ is defined as:

(2) $$\Phi_{r}=\Delta_{r}\phi_{r}$$

(3) $$\phi_{r}=\left\{ \begin{array}{l} \max(|R^{\vartheta_{-}}|,|R^{\vartheta_{+}}|)\;\mathrm{if}\;\min(|R^{\vartheta_{-}}|,|R^{\vartheta_{+}}|)\geq t_{s}\\ t_{s}\;\;\mathrm{otherwise}\; \end{array}\right.$$

where $\Delta_{r}$ is the weight coefficient, and $t_{s}$ is the size of the tiny object previously set.

Finally, the stability region corresponding to each clustering result is obtained and the stability map $M_{T}$ of the original image is generated. 

#### 3.2.2. Regional Saliency

The object has visual saliency in a certain area so that the human eye can often find it. RSS utilizes the local contrast mechanism to highlight salient areas and suppress uniform backgrounds. Firstly, the image is transformed into Lab space, and then the Gaussian filter is used to extract saliency information from the Lab color channel by low-pass filtering. The expression of two-dimensional Gaussian filter is:

(4) $$G(x,y,\sigma )=\frac{1}{2\pi \sigma^{2}}e^{-\frac{x^{2}+y^{2}}{2\sigma^{2}}}$$

where σ is the standard deviation of the Gaussian function.

The saliency of each pixel in the image is expressed by the difference between the Lab image and its Gaussian blur, and the saliency map obtained after filtering is normalized. The saliency map is expressed as:

(5) $$M_{A}=|(L,a,b)-(L_{G},a_{G},b_{G})|$$

where $L_{G}\;,\;a_{G}\;,\;b_{G}$ are the channel values of L , a , b after Gaussian blurring, respectively.

#### 3.2.3. Integration of Stability and Saliency

The stability and saliency results are integrated by pixel-by-pixel multiplication, denoted as $M_{T}\times M_{A}$. The saliency of the candidate regions extracted by stability detection is calculated and the saliency score is obtained to judge whether each candidate region is a real object. If the saliency score is greater than the average value of all candidate regions in the image, it is judged as a real object; otherwise, it is a false alarm.

### 3.3. STRSS Based on Visual Attention and Spatial-Temporal Information

RSS is only suitable for small object detection tasks with relatively simple and uniform backgrounds while having weak adaptability to complex backgrounds. Furthermore, there are a large number of false alarms and object missing problems for vehicle detection in mid-to-high altitude UAV images. Therefore, an improved vehicle detection algorithm (STRSS) based on visual attention and spatial-temporal information is proposed in response to the above problems.

#### 3.3.1. Road Segmentation

The background of mid-to-high altitude UAV images is relatively complicated. Most of the shooting scenes contain roads and the road area has the characteristics of uniform intensity and less interference. Considering the strong dependence relationship between the road and vehicles in the ground scene, we first extract the road area in the image by road segmentation networks to suppress the interference of complex backgrounds.

Most deep semantic segmentation networks are based on the encoder–decoder structure presently. The encoder is mainly responsible for feature extraction and context information capture, and the decoder predicts the labels of pixels through the decoded feature map. The network can capture semantic information of different scales by fusing the high-level and the low-level feature maps, which improves the robustness to multi-scale objects and recovers the loss of spatial information caused by the decline of resolution. At the same time, the encoder–decoder structure can better take into account the accuracy and efficiency of the network, making the end-to-end and pixel-level image segmentation methods become the mainstream. Some methods use Atrous Spatial Pyramid Pooling (ASPP) modules composed of convolutional layers of different atrous rates to expand the receptive field to capture multi-scale context information or recursive neural network to explore local dependencies to improve segmentation accuracy.

Deep semantic segmentation networks perform well in the road segmentation task of street view image, so they can be applied to the UAV scene to extract the road area from the mid-to-high altitude UVA image to generate a priori to guide the subsequent vehicle detection.

#### 3.3.2. Visual Stability Region Extraction

Candidate regions extracted by stability detection are the premise of saliency detection and the basis of tiny object detection. Therefore, the stability region extraction stage is further improved to obtain more effective candidate regions of objects.

(1) White objects processing

**Figure 3:**
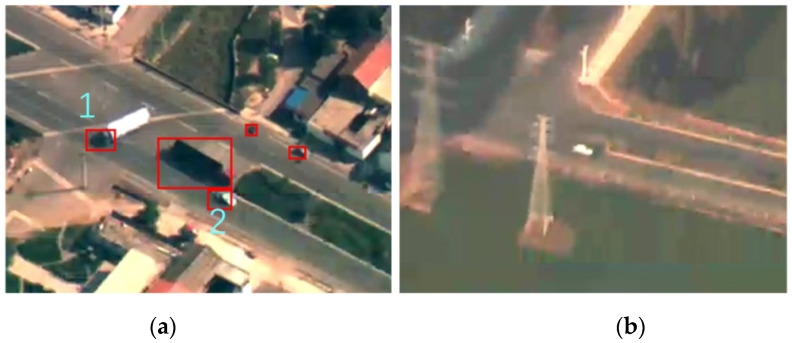
Example of missed white vehicles. Detection boxes 1 and 2 in (**a**) only detect the black cabin of the white truck or the black shadow around the white vehicle, while ignoring the real objects. (**b**) demonstrates thatthe only white object is not detected.

RSS believes that compared with the infrared scene, small objects obtained in the visual band usually have a smaller intensity value. However, white vehicles are typical objects in UAV images of mid-to-high altitude, whose intensity is greater than surroundings. RSS algorithm can achieve relatively accurate detection for black objects with low gray value, but will significantly miss white vehicles, as shown in Figure 3. 

Missing detection mainly occurs in the process of extracting the stability region. In the process of multi-level threshold segmentation, a set of thresholds with a grayscale range of [0, 255] and a step size of δ are used to perform binary segmentation on the grayscale image. The pixels with grayscale values less than the threshold are binarized into 255, and those with grayscale values greater than the threshold value are binarized into 0, and then the white connected regions are extracted from the black background. With the continuous increase of the threshold, the background area in the image becomes binarized into white, while the white object with a higher gray value becomes black after binarization and cannot be extracted. Therefore, we perform the inversion operation on the binary image with a high threshold according to a certain proportion to ensure that the connected region corresponding to the white objects can be extracted. 

Additionally, white trucks have black cabins, and some vehicles will form black shadows around them under certain lighting conditions, so there are always two overlapping detection boxes of the real object and its shadow in the final detection result. When clustering the low threshold binary image and the inverted high threshold binary image, clustering conditions are modified by doubling the centroid distance threshold to divide the object and its shadow into the same cluster, and expand the overlapping stability region, so as to obtain more precise detection results.

(2) Stability region discrimination based on Hu moment

After the second post-verification, the stability region of the object may correspond to a real vehicle or noise. To distinguish them, Hu invariant moment is used to constrain the shape of the binary stability region. 

Hu invariant moment is a statistical feature of an image, which has rotation, translation, and scale invariance. It contains seven eigenvectors constructed by the linear combination of second and third-order normalized central moments. 

Since the shape of the vehicle targets bear rectangular characteristics, the stability regions with irregular shapes, such as linear and triangular are removed by calculating the Euclidean distance between the invariant moment of each stability area and the feature vector of the rectangular template and taking into account each invariant moment component, so as to remove false alarms and improve the precision of detection.

#### 3.3.3. Spatial-Temporal Information Assistance

Due to the influence of environmental factors such as illumination, shadow, and occlusion, the stability detection results of different frames are inconsistent. This paper introduces the inter-frame motion relationship of the image sequence to assist the detection based on single-frame detection to improve the detection precision of tiny vehicles. 

The specific method is as follows: the idea of data association in multi-object tracking tasks is borrowed to associate the candidate regions in different frames so that the same vehicle in different frames in a certain period has the same ID. Then, the number of occurrences of the candidate region corresponding to each ID in the whole image sequence is respectively counted as a probability weighting factor. If a candidate region appears only once in the image sequence, it is more likely to be a false alarm, so its corresponding probability weighting factor is lower. Conversely, the candidate area with a higher probability of occurrence is more likely to be the real object. Therefore, the probability weighting factor is used as the weight of the subsequent saliency detection to further remove false alarms and improve the detection precision.

(1) Candidate region association

Spatial-temporal continuity means that the trajectory of the object in adjacent frames is continuous and the appearance change of it also has continuity in time. Therefore, regions in different frames are associated through the relative position relationship and appearance similarity of them. The position measurement is used to calculate the distance between the centroids of candidate regions, and the apparent similarity is measured if it is less than the threshold. The appearance measurement is mainly carried out by converting the original sub-image corresponding to the candidate region into Lab color space and performing color quantization. Each color channel is quantized to 4 bit, so the color number after quantization is reduced to $4^{3}$, which is used to calculate the Lab color histogram.

The formula for calculating the appearance similarity between two adjacent candidate regions is:

(6) $$Sim(R_{m}^{i},R_{n}^{j})=1-\alpha \chi^{2}[H_{m}^{i},H_{n}^{j}]$$

where $\chi^{2}[.]$ is the chi-square distance between the Lab color histograms, α is the weight of the chi-square distance, and $H_{m}^{i}$ represents the grayscale histogram of sub-image corresponding to the candidate region *m* in the ith frame.

Then, the KM matching algorithm is used to perform the candidate region association between adjacent frames, and the apparent similarity between them is used as the weight of the KM algorithm. The object association problem is transformed into the problem of finding the maximum weight perfect matching of the bipartite graph. Finally, the probability weighting factor corresponding to each ID is obtained.

The main task of this paper is not real object tracking, but only the use of probability weighting factors to modify the subsequent saliency detection within a certain time range, so the problems of the disappearance of old objects and the emergence of new objects are not considered.

(2) Locally weighted saliency decision

The saliency map is obtained after Gaussian filtering. In this section, we introduce a probability weighting factor to weight the Gaussian filtering results and calculate the final saliency score of the candidate regions extracted by stability detection. The stability regions corresponding to the same ID have the same probability weighting factors. The final saliency score is obtained to determine the authenticity of the candidate. If the saliency score is greater than the threshold, the candidate region is regarded as the true object otherwise, it is judged as a false alarm.

The saliency score is calculated as follows:

(7) $$S_{k}^{i}=P_{k}\sum_{(x,y)\in R_{k}^{i}}S(x,y)$$

where $R_{k}^{i}$ represents the ith stability region with the ID of *k*, $S_{k}^{i}$ represents the saliency score corresponding to $R_{k}^{i}$, and $P_{k}$ is the probability weighting factor of each stability region with the ID of *k*.

## 4. Experiments and Results

### 4.1. Data Collection

We collect a series of video sequences with RGB data based on actual flight data with a certain length of time under different atmospheric conditions, different flight altitudes, and imaging distances. The videos are shot in a plain area in eastern China, containing multiple different scenes such as cities, villages, and suburbs. Each scene is captured by the visible light and infrared integrated camera on the medium-altitude UAV. The imaging device has two degrees of freedom relative to the drone equipped with GPS (Global Positioning System), INS (Inertial Navigation System), and altimeter at the same time. The flying height of the UAV ranges from 3 to 7 km and the original resolution of each video frame is 1392 × 1040. 

Decoding, storage, automatic calibration and annotation of the collected remote sensing data are performed and a database of mid-to-high altitude UAV images containing video images and metadata is established.

### 4.2. Dataset Description

The vehicle detection algorithm proposed in this paper requires prior segmentation of the road area, so the mid-to-high altitude UAV image dataset we established is divided into road segmentation set and tiny vehicle detection set. The main information is shown in Table 1. The road segmentation set contains 413 original images with the size of 1392 × 1040, and two categories of road and background are manually annotated. Both original and binary label images are cropped into 512 × 512 for training on the Deeplabv3-plus network. 

In order to construct the vehicle detection dataset of mid-to-high altitude UAV images, we use LabelImg to annotate the vehicles. A total of 160 images in 4 scenes are labeled, of which there are 827 object instances with a size range of 10 × 10 to 50 × 50 pixels, including stationary vehicles and moving vehicles. To clarify, objects with 50 × 50 pixels are usually large trucks, while most other vehicles in images are only 10 × 10 pixels.

### 4.3. Experimental Settings

#### 4.3.1. Parameter Settings

RSS obtained a set of optimal parameters through a large number of experimental analyses. This paper makes fine-tuning on the basis of the original parameters to adapt to our mid-to-high altitude UAV image database. The step size of binary segmentation δ is set to 17.2, and the range of the number of pixels of stability region is set to [10, 2000] to ensure that our algorithm adapts to the changes of object size. The threshold of the second post-verification of the stability region $\Delta_{r}$ = 20, and the weight of the standard deviation of the Gaussian low-pass filter σ = 8.

#### 4.3.2. Evaluation Metrics

Both semantic segmentation and object detection can be regarded as a multi-classification task, and the confusion matrix can be used to compare the predicted output classification results with the truth. The difference is that semantic segmentation compares the predicted output results pixel by pixel, while object detection classifies the bounding box according to intersection over union (IoU) between the ground truth box and predicted box. Both of them can be evaluated by the precision, recall and F1 score. Furthermore, mean intersection over union (mIoU) is usually used to determine the accuracy of the segmentation.

(1) Precision

Precision represents the proportion of the true positive in the sample that the prediction is positive, which is defined as:

(8) $$Ρrecesion=\frac{TP}{TP+FP}$$

If the IoU between the predicted box and ground truth box is greater than the threshold, the detection box is true positive (*TP*), otherwise, it is false positive (*FP*). In addition, the ground truth box that does not match any prediction box is false negative (*FN*).

Recall indicates how many positive samples are correctly predicted, which is defined as:

(9) $$Recall=\frac{TP}{TP+FN}$$

(2) F1 score

F1 score is obtained by the weighted average of precision and recall, which is defined as:

(10) $$F1=\frac{2\times P\times R}{P+R}$$

(3) MIoU

MIoU is a commonly used evaluation metric for semantic segmentation, which calculates the ratio of intersection and union of two sets of real value and predicted value, and is defined as:

(11) $$MIoU=\frac{1}{k+1}\sum_{i=0}^{k}\frac{TP}{TP+FN+FP}$$

where *k* represents the number of categories. MIoU reflects the degree of coincidence between the predicted image and the real image. The closer the ratio is to 1, the higher the degree of coincidence and the higher the quality of semantic segmentation.

### 4.4. Experimental Results

#### 4.4.1. Evaluation of MTVD Algorithm

(1) Road segmentation

**Figure 4:**
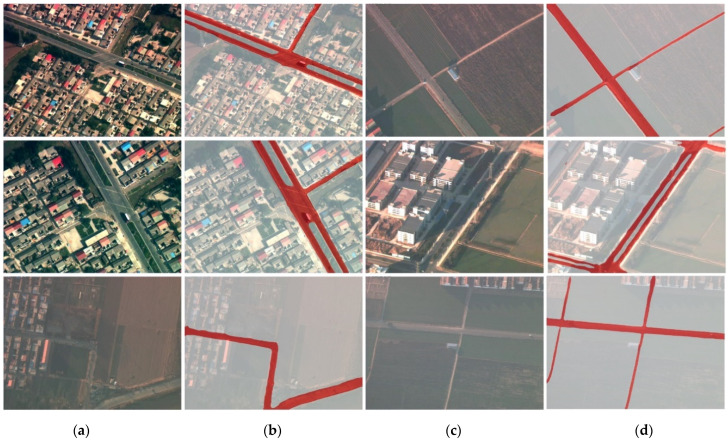
Visualization results of road segmentation. (**a**,**c**) Columns are the original images, (**b**,**d**) columns are the corresponding segmentation results, respectively, and the red markings represent the road area.

According to the UAV image and corresponding single-channel binary label, we train the semantic segmentation model based on Deeplabv3-plus [52], and we use the trained Deeplabv3-plus network to predict the images. Some of the prediction results are shown in Figure 4. The precision reached 92.69%, the recall reached 92.93%, the F1 score was 92.81%, and the MIoU was 87.18%. Experimental results prove that Deeplabv3-plus can effectively extract roads from mid-to-high altitude UAV images.

(2) Vehicle detection

The proposed algorithm based on the vehicle detection dataset of mid-to-high altitude UAV images is evaluated. In the mid-to-high altitude database we established, the quality of the image is greatly affected by weather, light, platform speed and altitude, and the surrounding environment, so the datasets have low contrast and poor clarity, and there are large background differences between different sequences. In addition, there are also certain differences between image backgrounds of different frames in the same scene, mainly including rotation, scaling, translation and other transformations due to the motion characteristics of UAV shooting, which further increases the difficulty of detection.

**Figure 5:**
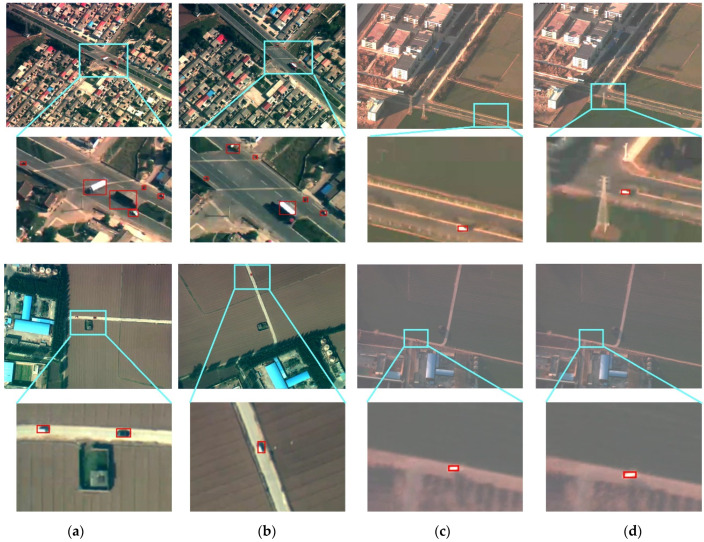
Visualization results of vehicle detection. (**a**,**c**) Lines show UAV images in different scenes. Part of the original image (cyan area) is enlarged and displayed in (**b**,**d**) lines accordingly to display the detection details.

Figure 5 shows the detection results of two different frames in four scenes. It can be seen that our proposed method can better realize the detection of limited pixel vehicles in the complex background of mid-to-high altitude UAV images. The comparison of the experimental results between RSS and our method is shown in Table 2.

#### 4.4.2. Ablation Experiments

**Figure 6:**
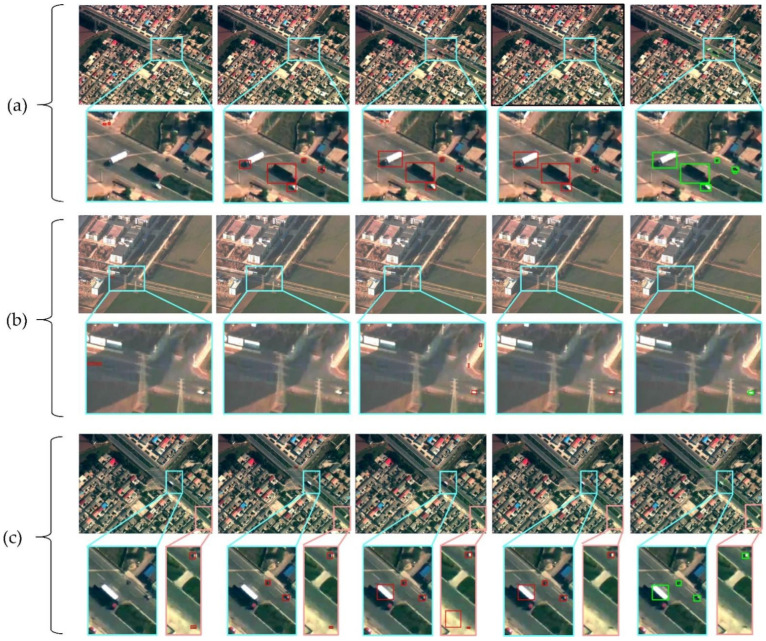
Visualization results of ablation experiments. (**a**–**c**) Respectively, represent three sets of examples, and the second line of each set shows the enlarged results (cyan or orange area). The first column shows the detection results of baseline, and the second column shows the results after fine-tuning parameters, modifying saliency decision conditions and removing the second post-verification. The third column shows the results after adding white objects processing, and the fourth column shows the results with the addition of Hu moment discrimination and locally weighted saliency decision. The fifth column displays the ground truth.

In this part, ablation experiments are performed to inspect the validity of each part. We take advantage of RSS as the baseline and fundamentally fine-tune the parameters to adapt itself to the characteristics of our dataset. Saliency detection is designed for removing false objects based on regional stability results, in other words, objects that are not extracted during the stability detection phase will never be detected. We attempted to improve the seriously missed detection and extract more effective candidate regions by modifying the condition of saliency decision, eliminating the second post-verification and white objects processing. However, at the same time, this will cause a decrease in precision. The methods of stability region discrimination based on Hu moment and locally weighted saliency decision are adopted to remove false alarms and improve detection precision. In this part, we conduct ablation experiments on each idea by changing one condition on the basis of the previous one while the other conditions remain unchanged. Figure 6 shows some of the subjective results of the above ideas in our approach.

We analyze the detection results with precision, recall and F1 scores. Due to the small size of the object, slight positioning deviation will lead to a significant decrease of IoU, and the detection box is closely surrounded by the object, while there is a certain error in the manual annotation. Moreover, due to the motion characteristics of UAVs and vehicles, the objects are prone to motion blur, which affects the accuracy of the location. Different from train-based methods, which can regression the accurate bounding box of the object, our method can only extract the object region according to the low-level feature of the image. For the above reasons, the threshold is set to 0.2 to obtain more reasonable evaluation results. The experimental results are shown in Table 3. The result we obtained may just be a local optimal solution, but the results of ablation experiments can still prove the effectiveness of each idea.

According to the results in Table 3, the precision of detection was improved by 27.33% by fine-tuning the parameter based on RSS, while recall was only improved by 4.35%. RSS considers the region whose saliency score is less than the average of all candidate regions in the whole image as the false alarm, which will lead to the problem that the real object is mistakenly removed. Therefore, we modified the saliency decision condition to set a fixed threshold, and the recall increased by 18.02% while precision was increased by 3.82%. 

The motion characteristics of UAVs and vehicles, as well as environmental factors, lead to no clear boundary between vehicle and background, and the stability region second post-verification leads to the removal of many real objects. Experimental results show that the application of removing second post-verification can help significantly improve the recall without any false alarms. Then, we dealt with white objects by inversion and clustering after which the recall increased by 20.56%, but the precision decreased by 6.22% due to the introduction of white noise during processing.

Finally, Hu moment discrimination and locally weighted saliency decision were used to further remove false alarms, and the optimal detection effect was obtained. The precision increased to 71.26%, the recall increased to 86.94% and the F1 score reached 78.32%. 

#### 4.4.3. Tinier Object Exploration

In this part, original images are resized to generate series of images with lower resolution, and the size of the vehicles is reduced proportionally. Additional test images are used to explore the detection effect on tinier objects. Since our proposed algorithm is pixel level, the bilinear interpolation method used in scaling will change the pixel value and affect the detection result to a certain extent. During the test, we appropriately modify the parameters related to image resolution and object size, and send low-resolution images into the detection model. The detection results obtained are shown in Table 4.

It can be seen from Table 4 that the detection accuracy reduces slightly with the decrease of resolution. After three times down-sampling of the original image, the object size of 10 × 10 in the original image is only about 3 × 3, and the F1 score is 73.83%. Compared with the original size, it only decreases by 4.49%, which is acceptable within a certain range. The proposed algorithm mainly uses the gray difference between the object and background for detection in the resolution of the input image, so the detection results are only related to the pixel value and have nothing to do with the size of objects. The experimental results show that the proposed algorithm can still effectively detect the limited pixel objects below 10 × 10 pixels. 

We also compare our approach with the current state-of-the-art object detection networks, such YOLOv5, on the scaled mid-to-high altitude UAV dataset of different sizes. F1 scores obtained by fine-tuning Yolov5 network on our dataset are compared with the results of our method in Table 5. According to Table 5, our method performs worse than YOLOv5s and YOLOv5m on the original images and the images down-sampled by a factor of two thirds, but performs better when the scaling factors are one-half and one-third. Our method achieves 2.27% higher F1 score than YOLOv5s and 2.66% higher than YOLOv5m after twice down-sampling of original images, and 4.1% higher F1 score than YOLOv5s and 3.27% higher than YOLOv5m after triple down-sampling. Experimental results demonstrate the advantage of the method we put forward on tiny objects with limited pixels blow 5 × 5 compared to the deep network. Although our method is slightly inferior to YOLOv5 in larger object detection, it has stronger robustness and superior generalization performance for object size.

## 5. Conclusions

This paper brings forward a tiny vehicle detection approach based on visual attention and spatial-temporal information for mid-to-high UAV images. On account of the scene dependence of vehicles, we segment road areas of the image through the deep semantic segmentation network and extract the stability regions from road areas as candidate regions of the vehicles. To remove the false alarms, we introduce the inter-frame motion relation and design a locally weighted saliency decision method to perform the second inspection on the candidate regions. Experimental results demonstrate that our method achieves effective detection of vehicles in mid-to-high altitude UAV images. The detection algorithm does not require large-scale annotation data for model training. Moreover, our method is more robust to the size of objects and performs well in the detection of vehicles with limited pixels below 5 × 5, which can effectively provide support for image data analysis of ground.

## Figures and Tables

**Table 1:** Main information of the mid-to-high altitude UAV image dataset we established.

Dataset	Road Segmentation Set	Tiny Vehicle Detection Set
Number of images	413	160
Image size	1392 × 1040	1392 × 1040
Annotated category	road and background	vehicle
Number of objects	-	827
Size of objects	-	10 × 10–50 × 50

**Table 2:** Comparison of the results between with RSS and our method.

Method	Precision (%)	Recall (%)	F1 Score (%)
RSS	36.77	16.81	23.07
ours	71.26	86.94	78.32

**Table 3:** Ablation experimental results. Only a new idea is added on the basis of the previous experiment while the other conditions remain unchanged.

Newly Added Conditions	Precision (%)	Recall (%)	F1 Score (%)
Baseline	36.77	16.81	23.07
Fine-tuning the parameters	64.10	21.16	31.82
Modify saliency decision condition	67.92	39.18	49.69
Remove second post-verification	69.55	69.04	69.30
White objects processing	63.33	89.60	74.21
Hu moment discrimination	68.35	83.56	75.19
Locally weighted saliency decision	71.26	86.94	78.32

**Table 4:** Results of different resolutions.

Scaling Factor	Size of Usual Objects	Precision (%)	Recall (%)	F1 Score (%)
1	10 × 10	71.26	86.94	78.32
2/3	7 × 7	70.85	80.53	75.38
1/2	5 × 5	70.91	78.11	74.34
1/3	3 × 3	72.17	75.57	73.83

**Table 5:** Comparison of our method with YOLOv5 on our dataset.

Method	Scaling Factor	1	2/3	1/2	1/3
	Size of Usual Objects	10 × 10	7 × 7	5 × 5	3 × 3
YOLOv5s	F1 score	83.87	83.62	72.07	69.73
YOLOv5m	F1 score	85.41	83.61	71.68	70.56
Ours	F1 score	78.32	75.38	**74.34**	**73.83**

## Data Availability

The data are not publicly available due to the privacy of the dataset.
